# Origin of tensile strength of a woven sample cut in bias directions

**DOI:** 10.1098/rsos.140499

**Published:** 2015-05-13

**Authors:** Ning Pan, Radko Kovar, Mehdi Kamali Dolatabadi, Ping Wang, Diantang Zhang, Ying Sun, Li Chen

**Affiliations:** 1 Department of Biological and Agricultural Engineering, University of California, Davis, CA 95616, USA; 2 Department of Textile Technology, Technical University of Liberec, Halkova 6, Liberec 46117, Czech Republic; 3 Institute of Textile Composites, Tianjin Polytechnic University, Tianjin 300160, People’s Republic of China

**Keywords:** fabric strength, bias directions, yarn pullout and breakage, failure types, sample width effect

## Abstract

Textile fabrics are highly anisotropic, so that their mechanical properties including strengths are a function of direction. An extreme case is when a woven fabric sample is cut in such a way where the bias angle and hence the tension loading direction is around 45° relative to the principal directions. Then, once loaded, no yarn in the sample is held at both ends, so the yarns have to build up their internal tension entirely via yarn–yarn friction at the interlacing points. The overall fabric strength in such a sample is a result of contributions from the yarns being pulled out and those broken during the process, and thus becomes a function of the bias direction angle *θ*, sample width *W* and length *L*, along with other factors known to affect fabric strength tested in principal directions. Furthermore, in such a bias sample when the major parameters, e.g. the sample width *W*, change, not only the resultant strengths differ, but also the strength generating mechanisms (or failure types) vary. This is an interesting problem and is analysed in this study. More specifically, the issues examined in this paper include the exact mechanisms and details of how each interlacing point imparts the frictional constraint for a yarn to acquire tension to the level of its strength when both yarn ends were not actively held by the testing grips; the theoretical expression of the critical yarn length for a yarn to be able to break rather than be pulled out, as a function of the related factors; and the general relations between the tensile strength of such a bias sample and its structural properties. At the end, theoretical predictions are compared with our experimental data.

## 2. Introduction

In comparison with rigid solids, textile fabrics are highly complex materials for mathematical formulation and treatment. A major portion of the complexity is attributable to direction-dependence, i.e. the anisotropy of the fabric structure. In the case of woven fabrics for example, the anisotropy is originated chiefly by the fact that the fabric is formed by two groups of yarns (warp and weft) interlaced perpendicularly. As a result, the strength tested along the warp or weft directions (the principal directions) will diverge if the two groups of yarns are not identical. Even if the strengths in both principal directions are the same when the same types of yarns and weave structure are used, the strengths tested at directions other than the principals (the bias directions) will still be very different. Although this anisotropy imparts huge benefits (e.g. draping and shape conformity, to just name a couple) to woven fabrics in their intended purposes for clothing and home furnishings, it complicates, nevertheless, any analytical attempts dealing with fabric properties.

**Figure 1.**
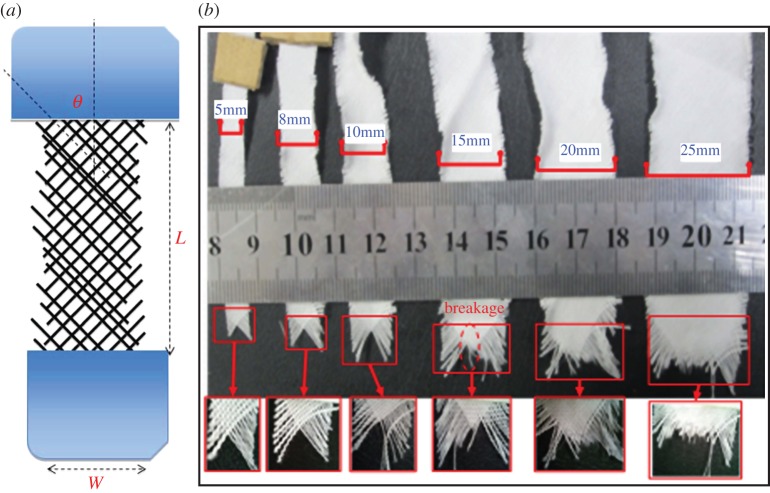
(*a*) Fabric sample tested in the bias directions. (*b*) Examples of broken samples of different widths.

Textile structures are interesting engineered objects, for they acquire their strengths via, entirely or partly, their internal frictions [1,2]. In the case of woven fabric, this interyarn friction is brought into play by the interlacing between the warp and weft yarns [1,3,4]. Without such interweave of the yarns, a woven fabric is disintegrated into merely a sheet of two layers of yarns disjoined from each other. Of course, both layers of parallel yarns still possess strength, but only when loaded along the respective yarn axis. Once woven, however, a fabric acquires the load carrying capacities not only along the two perpendicular principal directions coinciding with the warp and weft yarn axes, but also along any other bias directions, thus translating a two-axes-loading-only system into an omnidirectional material which is able to carry axial tension and even in-plane shear loads. Of course, those tensile and shear strengths, such as other properties, depend on the relative alignment of the load to the principal directions—thus, the behaviour of anisotropy. Additionally, the fabric in-plane shear properties are closely related to its tensile properties, mainly because yarns in a fabric fail predominantly in tension, regardless the nature of the external loads [1]. An associated behaviour in such fabrics is the ability for yarns to reorient themselves towards the loading direction and such a realignment ability of yarns is enabled again by the frictional interconnection between the warp and filling yarns—*i.e. the friction provides both connectivity and mobility to the entire structure.* The friction can even incite phase change in fabrics under large deformation—fabric buckling and yarn jamming, the *phenomena where fabric turns from an ordered phase into a disordered one*. Another interesting case is when a fabric sample is cut in such a way where the bias angle and hence the loading direction is at 45° to the principals. Then, once loaded, no yarn is held at both ends, as shown in figure 1. The yarns now have to build up internal tension entirely via yarn–yarn friction at the interlacing points. In other words, the yarn–yarn interactions have turned to be the only mechanism for the sample to attain strength as a whole. Compared with simple shear and uniaxial extension tests, uniaxial bias extension test can capture more information about the fabric mechanics, and in fact a thorough investigation on such bias tensile behaviour of woven fabric will involve all the major issues mentioned above.

From the public record, Kilby [5] was the first to study in detail the anisotropic mechanics of woven fabrics, and at about the same time Cooper [6] carried out a series of bias extension tests under a cycling low load to analyse the response of woven fabrics. There has been a long list of research activities reported on the topic since. Spivak [7] attempted to find the connection between bias extensions and shear properties for plain-weave fabrics, and concluded rather un-encouragingly that as different fabrics behave so distinctively it is unlikely that a universal relationship between the two groups of properties can be established. More specifically however, Buckenham [8] discovered that the significant differences between the fabric shear test and the bias extension test were caused mainly by the occurrence of the sample buckling in the shear test, and for fabrics that did not buckle during shear test, there was a highly linear correlation in sample shear rigidities derived, respectively, from the two types of tests.

Further, Du & Yu [9] used the Spivak model [7] to correlate the bias extension test operated on an Instron-type system with the shear test from KES-F1 instruments. It was concluded that bias extension in 45° direction exhibited the highest correlation (over 0.8) with its shear behaviour, compared with those in other directions, confirming that reported earlier by Spivak & Treloar [10]. Naujokaityte *et al.* [11] also investigated the 45° bias extension behaviours of finished as well as unfinished cotton fabrics, captured the greyscale high-resolution digital images of the deformed sample during uniaxial tension process and analysed the influence of different parameters on the fabric responses in bias extension tests. Bekampiene & Domskiene [12] studied the influence of aspect ratio of fabric specimens on bias extension behaviour. Dolatabadi & Kovar [13] used two-dimensional fast Fourier transform to assess the exterior position of yarns in deformed fabrics, and reported that there was a critical shear angle related to fabric geometrical size. Dridi *et al.* [14] developed an orthotropic hyperelastic model to simulate the bias extension test, and their numerical analyses found that the ratio of shear and tensile rigidities of fabrics is a critical factor for shearing deformation. Harrison *et al.* [15] put forward a novel biaxial and bias extension test to characterize wrinkling and shear–tension coupling behaviours for woven fabrics. Meanwhile, Pan and co-workers [16,17] examined the connection between shear and bias tensile strengths of woven fabrics and proposed a general failure criterion for the materials. A thorough review on the experimental measurement of fabric mechanical properties can be found in Bassett *et al.* [18].

Using textile fabrics as reinforcement, the questions in fabric mechanical behaviours in bias direction have attracted enormous interest from composite area as well. Peng & Cao [19] adopted a dual homogenization method combined with finite-element approach to develop a pin-joint unit cell model, simulating a trellising test which is deemed the major deformation mechanism in the bias extension. Potter [20] studied the behaviour of woven reinforcements under bias extension also using the pin-joint net assumption, and proposed a theoretical analysis to predict the global behaviour of unidirectional prepreg. Then, Lebrun *et al.* [21] compared both bias-extension test and the picture-frame method, developed theoretical equations based on the pin-joint net approximation and proposed a modified version of picture-frame test to explore the influence of the fabric architecture on the material properties. Again, Peng & Cao [22] proposed a non-orthogonal constitutive model, thus decoupling the shearing and tensile deformations, to characterize the anisotropic properties of plain-weave composites under bias extension and shear load. They then used the stress and strain transformation between the global and local coordinates to develop constitutive equations for woven composite. Yu *et al.* [23] focused on the large deformation mechanism during the bias extension tests carried out under different temperatures and loading speeds. Then, digital image correlation analysis was used to identify three deformation phases, and at the end, a theoretical model based on energy method was proposed to explain the failure mechanisms.

In this paper, we take a different approach from the existing literature and deal with the fracture physics at micro and local level during fabric failure by focusing on the mechanisms of how the individual yarn strength is translated into fabric strength via interyarn friction. It is the first complete study in exploring the origin of the tensile strength of a narrow and bias woven fabric via theoretical analysis and experimental validation.

Beginning with an experimental work conducted on three fabrics, we first examine the fractured samples and explore the important parameters involved in the bias woven fabric samples of increasing width. Then, by differentiating the contributions from both broken and pulled out yarns as functions of sample width, we build a theoretical model to describe the sample fracture process based on the probabilistic strength theories, and thus predict the sample strength at different levels of the related parameters. The predictions are then compared with the experimental data, along with discussions on some key issues.

The usefulness of this study is multiple. First, for a narrow bias woven fabric sample, this is the first such study on the origin of its tensile strength, both theoretically and experimentally. Furthermore, the conclusions drawn can be helpful in understanding the mechanics of fabric-reinforced composites. Although it seems once the fabric as the reinforcement is embedded into a matrix system, the fabric frictional shear behaviours discussed in this work are restricted or even eliminated and its structural characteristics are reduced into something representable by a single fibre volume fraction. However, given the complexity of the composite behaviours, such a view is an over-simplification with no supporting evidence. After all, regardless how the fabric is interacting with the matrix, we are still dealing with a composite system where both the reinforcement and matrix contribute collectively to the system behaviours. For instance, failure of composite is known as a complex and multistage process involving the so-called fragmentation phenomenon [24], where the larger reinforcement breaks down to small pieces, yet still carrying load and contributing to the overall behaviours of the system: our analysis on a small bias sample is thus relevant to the understanding of the composite behaviours.

More importantly, this experimental and modelling work actually presents a methodology dealing with the strength of a discrete system formed by components whose tensile strengths are given. In other words, this work shows the fracture mechanics of a discrete system formed by individual components connected only with contact frictions. Our study demonstrates that in translating the strengths of the components into the system performance, the probabilistic distributions among the constituents have to be included.

## 3. A fabric bias tensile experiment

Three kinds of plain-weave fabrics as shown in table 1 have been collected for this study. For the bias tensile tests, our experimental work was conducted on the fabric samples cut in such a shape as in figure 1*a* that, when loaded, no yarn is held by both ends, so that all the yarns build up stress entirely via yarn–yarn friction at the interlacing points. As a result, only the yarns with enough embedded length can reach the stress level high enough to break, whereas the shorter yarns can only be pulled out, contributing much less towards fabric strength. In preparing the samples, both bias direction angle *θ* from the warp direction and the sample length *L* were fixed at values *θ*=45° and *L*=100 mm. The sample width, however, was allowed to change at most in eight steps of 5, 8, 10, 15, 20, 25, 35 and 45 mm so as to examine how the sample width, thus different failure mechanisms or types, impacts the sample strength.

**Table 1.** Parameters of three kinds of fabrics.

	thread count, cm^−1^		yarn size, tex		no. filaments		
fabric type	warp	weft	warp	weft	warp	weft	areal density, g m^−2^
polyester/cotton	22	22	24	24	—	—	108.2
wool	32	30	30	25.5	—	—	132.5
glass	24	24	21.7	21.7	160 + 15	160 + 15	108.1

For our test, the standard test method ASTM D 5035-11 was followed whenever possible, with stated adjustment in sample width. Dynamometer Instron 4411 (AGS material tester) was used with load cell capacity of 500 N and pneumatic jaws. Sample gauge length was *L*=100 mm and the crosshead speed 1 mm s^−1^. Fabric pretension was set as zero. Each test result in this study is the mean of three repeats.

Figure 1*b* shows examples of broken samples of different width for the glass fabric. Because all yarns were held only at one end during testing, so that when the sample width is very small (*W*=5 mm, for instance in figure 1*b*), no yarn has sufficient length to realize its full strength via the friction, and thus was pulled out, leading to a low overall specimen strength and a broken specimen cross section of isosceles triangle shape—the space left by the pulled out yarns in specimen. Yet, as the width increases, growing number of yarns at the centre break, and only the short yarns at the fringes are still pulled out: the broken specimen cross sections evidently illustrate that the tip of the once triangle becomes increasingly blunt or flat.

**Figure 2.**
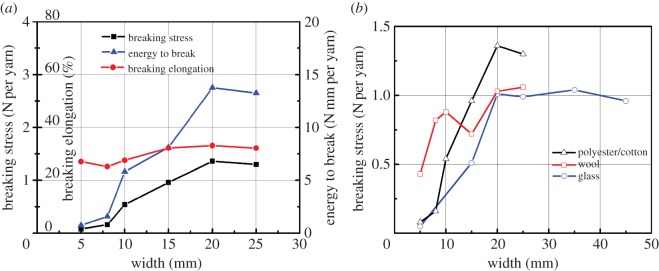
(*a*) Results for polyester/cotton fabric in bias tests. (*b*) Influence of sample width on tensile strengths.

All the testing data as specified in the standard test method ASTM D 5035-11 are provided in tables 2–4, and are also plotted against the sample width in figure 2. For clarity, figure 2*a* shows only the data for the polyester/cotton fabric. Both fabric strength (N per yarn) and breaking elongation increase from a lower level for narrower specimens along with sample width *W*, but stabilize when specimen width reached some critical values, and then levels off or drop a little, when *W* further increases to *W*=25 mm (it is suspected that as the sample becomes too wide, gripping over the sample width becomes less uniform, leading to the slight drop in the results which otherwise would be a plateau). Such width effect is more apparent on fabric strength than on its breaking elongation, whereas the breaking energy—being a joint product of both strength and elongation, follows and yet magnifies the trend. In other words, for such a bias test, unlike the orthogonal strip test, sample width *W* becomes an important variable. Figure 2*b* compares all three fabrics but only in fabric strengths and similar trend is clearly exhibited.

**Table 2.** The bias tensile breaking load of fabrics (*L*=100 mm).

	load, N								
fabric type	width (mm)	5	8	10	15	20	25	35	45
polyester/cotton		1.39	4.44	17.40	43.96	87.03	104.05	—	—
wool		10.24	29.62	38.60	49.07	93.00	116.55	—	—
glass		0.85	—	—	24.07	62.78	76.99	113.33	134.12

**Figure 3.**
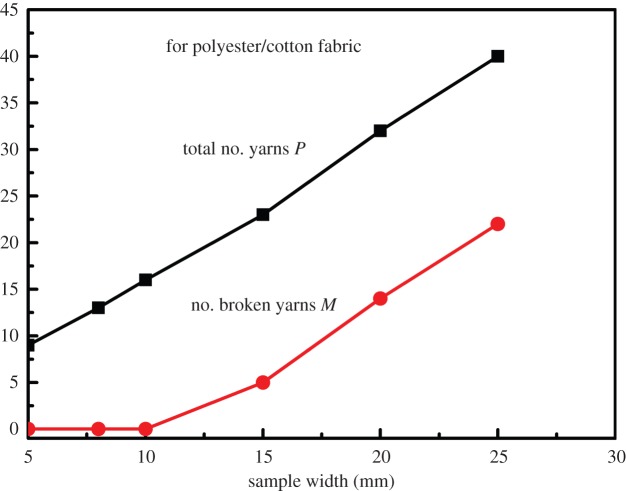
Numbers of broken and total yarns versus width.

Figure 3, using data in tables 5–7, helps explain this altering failure types by showing the changes of total yarn numbers *P* and broken yarns *M* versus the specimen widths *W*, where the total number *P*=*M*+*N* and *N* is the yarns being pulled out (slipping). Practically all yarns are pulled out at the beginning when *W*=5 mm, so that *M*=*P* and *N*=0. As the specimen width *W* and thus the total number *P* rise, growing number of yarns is broken, leading to the increase in sample strength. It is interesting to note that as specimen width *W* and total yarn number *P* grow, the number of slipping yarns *N*, indeed, stays nearly constant, confirming that the yarns being pulled out are always the ones at both edges, roughly a constant and independent of sample width *W*.

**Table 3.** Elongation at break under bias tensile loading (*L*=100 mm).

	elongation at break, %								
fabric type	width (mm)	5	8	10	15	20	25	35	45
polyester/cotton		27.06	25.22	27.54	32.23	33.14	32.19	—	—
wool		26.67	34.63	31.90	29.23	30.09	33.09	—	—
glass		19.72	—	—	22.98	25.14	23.67	25.17	28.20

**Table 4.** Energy to break under bias tensile loading (*L*=100 mm).

	energy to break, N mm yarn^−1^								
fabric type	width (mm)	5	8	10	15	20	25	35	45
polyester/cotton		0.73	1.58	5.82	8.11	13.75	13.25	—	—
wool		5.96	13.22	11.88	8.91	15.99	18.06	—	—
glass		19.72	—	—	22.98	25.14	23.67	25.17	28.20

**Table 5.** Yarn number as function of fabric width *W* for polyester/cotton fabric.

*W* (mm)	5	8	10	15	20	25
total	9	13	16	23	32	40
slipping	9	13	16	18	21	20
broken	0	0	0	5	11	20

**Table 6.** Yarn number as function of fabric width *W* for wool fabric.

*W* (mm)	5	8	10	15	20	25
total	12	18	22	34	45	55
slipping	12	17	18	18	15	13
broken	0	1	4	16	30	42

**Table 7.** Yarn number as function of fabric width *W* for glass fabric.

*W* (mm)	5	15	20	25	35	45
total	8	24	31	39	54	70
slipping	8	24	29	31	35	41
broken	0	0	2	8	19	29

Clearly, the major variables, in this case, include the bias direction angle *θ*, the sample (gauge) length *L*, and sample width *W*. When *θ*=0° or 90°, the case reduces to the orthogonal ones. Yet, for a given *θ* of other values

— if *W* is too small, all yarns will slip and be easily pulled out and the fabric strength is minimum;
— when *W* increases, more and more yarns can realize their strength and break, and the fabric strength increases.

When both *θ* and *W* are given, however,

— when sample length *L* is zero, all the yarns are held in both ends, and are able to break;
— as *L* increases, however, more yarns slip, depending on the values of *W* and *θ*.

Thus, the issues to be dealt with in modelling this bias sample strength include:

— the exact mechanism and details how each interlacing point imparts the frictional constraint for yarns to acquire tension to the level of their strength, when both yarn ends were not actively held by the testing grips;
— next, the theoretical expression for the critical length for a yarn to be able to break rather than being pulled out, as a function of bias direction angle *θ*, sample width *W* and length *L*, as well as other parameters; and
— as yarns can only be broken in tension, the yarns in a fabric sample tested in bias direction have to realign themselves to be stretched so as to break or be pulled out. Then, how such yarn realignment influence the fabric behaviour and hence its strength.

## 4. Theoretical considerations

In fact, the issue of yarn realignment is most significant in fabric tear test, and there have been some comprehensive studies devoted to the subject [25,26]. It will hence be considered negligible in our analysis. In addition, the influences of the bias direction angle *θ* and the sample length *L* have been investigated extensively [5,16,27,28]. Our study will hence focus on the impact of sample width *W*, first because it has rarely if ever been dealt with in existing public literature, and also that just changing the sample width *W* alone is a subject with sufficient challenges, as demonstrated below.

### 4.1 The sample loading and geometries

**Figure 4.**
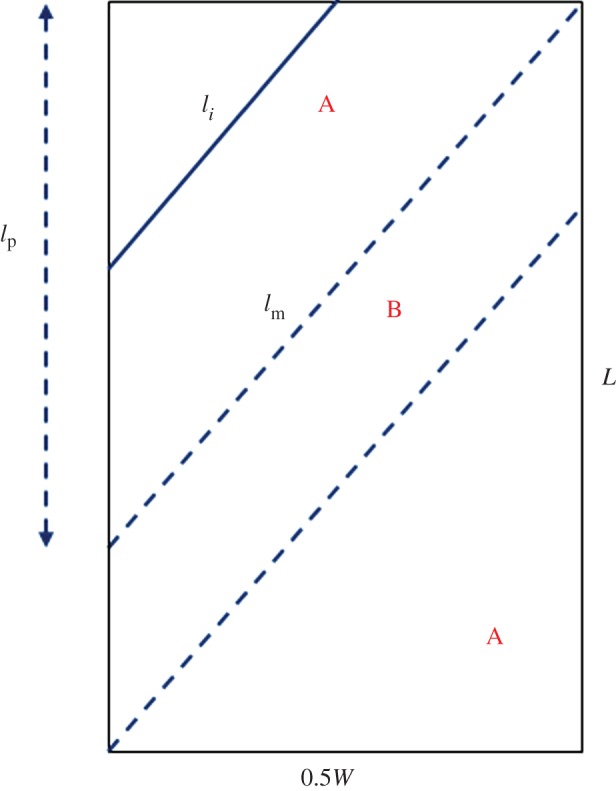
One half (0.5*W*) of the bias sample for analysis.

From the illustration in figure 1, it is seen that a bias sample is still symmetrical about the centreline in the warp direction, so we need to analyse only one-half (0.5 *W*) of the sample as sketched in figure 4. In the part shown in figure 4, the two lines of maximum yarn length *l*_m_ divide it into three parts: in between the two *l*_m_ lines is the region B where yarns are all with the same length *l*_m_, and thus is the strongest region. As a result, the sample strength is actually determined by the other two equal regions A with mixture of longer and short yarns: analysing either of them (e.g. the top left one) will give us the sample strength.

Next, we need to find out in the region A the numbers of yarns that during the test will be broken and pulled out, respectively, at a given width *W*, and the sum of total forces from both groups will give us the total breaking load of the sample. By definition, the total number of yarns in the width direction for the half sample width is

4.1 $$P=\frac{0.5Wn_{y}}{\sin\theta },$$

where *n*_y_ is the fabric count—number of yarns per cm fabric length. Thus, the spacing between two yarns in this bias sample is sin *θ*/(*n*_y_). Other simple geometries in figure 4 include *l*_m_=0.5*W*/sin *θ* and the sample length along the width direction at *i*th yarn, *l*_p_=*i*/(*n*_y_⋅ sin *θ*). The embedded length *l*_*i*_ for yarn *i* (*i*=1 to *P*) becomes

4.2 $$l_{i}=\frac{i}{n_{y}\text{sin}\theta }.$$

As expected a yarn with an embedded length less than a critical length *l*_c_ (yet to be determined) will slip or be pulled out, so that the total number of slipping yarns is

4.3 $$N=\{k\},l_{k}<l_{c}\;\text{and}\;l_{k+1}\geq l_{c},$$

and the remaining *M* number of yarns will break, where

4.4 $$M=P-N=\frac{0.5Wn_{y}}{\text{sin}\theta }-N.$$

The sample tensile strength is a result of both yarn breakage and pullout in a sample cut in bias directions. Note that because the embedded length *l*_*i*_ for yarn *i* increases with sample width *W*, the number of broken yarns *M* for a constant critical length *l*_c_ grows with *W* as shown in equation (4.4) and by the experimental results in figure 3.

### 4.2 Mechanics at an interlacing point and yarn pullout from fabric

The next step is to find out the force carried by a yarn being pulled out of the fabric as a function of embedded yarn length *l*_*i*_ until the length exceeds the critical value *l*_c_. The key to the answer lies in the interaction at the interlacing points between warp and weft yarns. Assume this resistance constraint *τ*_y_ per crossing point consists of two parts, a compression-dependent (frictional) part and a compression-independent (adhesive) one [4], i.e.

4.5 $$\tau_{y}=\tau_{y1}+\tau_{y2}.$$

*For plain weave* and equal warp (*L*) and weft (*T*) parameters, i.e. yarn thickness *t*_y*L*_=*t*_y*T*_=*t*_y_, fabric count *n*_y*L*_=*n*_y*T*_=*n*_y_, we have for the compression-dependent component from [4]

4.6 $$\tau_{y1}=\frac{2\mu }{C_{y}}(\sigma_{L}+\sigma_{T})\text{sin}[\arctan(4t_{y}n_{y})].$$

It is obviously generated from the tensile load exerted either uniaxially as in the warp direction *σ*_*L*_ (weft direction *σ*_*T*_=0), or biaxially with both *σ*_*L*_ and *σ*_*T*_ to cause a tightening effect to the fabric so as to increase the pressure at the yarn contact points. Here *σ*_*L*_ and *σ*_*T*_ are the stresses in the warp and weft directions; *C*_y_ represents the length of the yarn–yarn contact area; *μ* is the yarn–yarn contact frictional coefficient.

The adhesive component is a non-zero quantity even when the sample is load free and it satisfies [4]

4.7 $$\tau_{y2}=\frac{2\gamma_{s}w_{y}}{C_{y}\rho }\tanh(2\rho w_{y}),$$

where *w*_y_ is the yarn width, *γ*_*s*_ is the adhesive resistance at the contact points when compression is zero, and *ρ* is a factor reflecting the yarn tightness with a unit of length^−1^:

4.8 $$\rho =\frac{1}{t_{y}}\sqrt{\frac{G_{y}}{\pi E_{y}}},$$

where *G*_y_ and *E*_y_ are the shear and tensile moduli of the yarn, respectively. Thus, the total unit constraint *τ*_y_ can be expressed as

4.9 $$\tau_{y}=\frac{2\mu }{C_{y}}(\sigma_{L}+\sigma_{T})\text{sin}[\arctan(4t_{y}n_{y})]+\frac{2\gamma_{s}w_{y}}{C_{y}\rho }\tanh(2\rho w_{y}).$$

That is, even where *σ*_*T*_=0, as long as a yarn is pulled out from the fabric structure, the associated tension *σ*_*L*_ will generate at each contact the point resistance *τ*_y**1_. When *τ*_y_ is high enough, the yarn will break.

Once we obtained the resistance *τ*_y_ at an interlacing point, we can then proceed with next issue, i.e. the pulling out force on *N*=*P*−*M* yarns that slip during extension. Clearly, this pulling out force will be a function of embedded yarn lengths. There has been some research on this issue [29–32], and we use here the results by Pan & Yoon [31]. The major assumptions corresponding to the theory are listed below:

(a) assume that the interactions between the warp and weft yarns will not affect the properties of individual yarns—we will, however, lift this assumption in the next section;
(b) neglect the jamming effect and distortion of woven fabrics during the process.

Based on the analysis in [31], the maximum force *P*_m_ to pull a yarn over a single crossing point with resistance *τ*_y_ in a woven fabric is clearly

4.10 $$P_{m}=\tau_{y}.$$

Then, for a yarn with embedded length of *l*_y_, the maximum tensile force required to pull this yarn out of the fabric can be calculated as

4.11 $$P_{lm}=\tau_{y}\text{Round}[n_{y}l_{y}],$$

where the Round function Round() is used to round the fraction part to reflect the discrete nature of the crossing points. This and equation (4.9) show that for a given embedded yarn length *l*_y_, the pullout force *P*_*l*m_ is a function of fabric count, yarn shape, dimension and mechanical properties. In the critical case when the pullout force reaches the yarn breaking load *P*_y*u*_

4.12 $$P_{lm}=\tau_{y}\text{Round}[n_{y}l_{y}]=P_{yu},$$

the yarn will be broken in the fabric. Equation (4.12) thus sets the criterion for identifying the number of broken yarns *N* using equation (4.3). The only issue remains in the equation is what yarn breaking load *P*_y*u*_ to use in equation (4.12). As the yarns are already integrated into a structure, will they still possess the same originally determined *ex situ* yarn strength? In other words, will the yarn–yarn interactions inside a fabric alter that original *ex situ* yarn strength? To answer this question, we have to examine the effect on yarn strength owing to interactions between yarns in a fabric, and thus remove the first assumption listed above, originally adopted in [4,31].

### 4.3 Interactions between yarns during sample extension—the yarn *in situ* behaviour

To understand the effect of yarn–yarn interactions on yarn/fabric strengths, we cite here an earlier work by Shahpurwala & Schwartz [3] where they studied the relationships between strengths of individual yarns, yarn bundles and fabrics. In the study, they validated or revealed some connections on the tensile strengths between fabric and its constituent yarns.

(a) The statistical mean strengths of yarn bundles are no greater than those of their constituent yarns, $\bar{\sigma_{b}}\leq \bar{\sigma_{y}}$. In other words, as the individual yarns form a bundle, they collectively weakened themselves owing to the load sharing between the broken and still-surviving yarns in the bundle, leading to this bundle strength deterioration. However, the authors provided only theoretical predictions, with no experimental yarn bundle data in their study.
(b) The mean fabric uniaxial strengths are no smaller than that of the constituent yarn bundle in the same direction $\bar{\sigma_{F}}\geq \bar{\sigma_{b}}$. That is, when yarn bundles (warp and weft) were interlaced in perpendicular directions to form the fabrics, the whole system is reinforced. Clearly, what accounts for the strength discrepancy between the parallel yarn sheet and a real fabric is the yarn interaction occurring in the interlacing points.
(c) The mean fabric strengths $\bar{\sigma_{F}}$ can be smaller than, equal to or greater than $\bar{\sigma_{y}}$ of its yarns.

To more thoroughly analyse the phenomenon involved, we have to briefly review the studies on statistical nature of fibrous material strength and the associated theories. It has become common knowledge that individual yarn samples taken from the same package will not yield the same tensile strength because of the various statistical variations over yarn length, as stipulated by Peirce in 1926 [33]. In fact, according to Coleman [34], the cumulative probability distribution function of such sample population is of the Weibull type, and hence the mean or the expected value of the yarn breaking strength, $\bar{\sigma_{y}}$, can be calculated as

4.13 $$\bar{\sigma_{y}}={\left(l_{y}\alpha \right)}^{-1/\beta }\Gamma \left(1+\frac{1}{\beta }\right),$$

where *α* is the scale parameter and *β* the shape parameter, both independent of sample length *l*_y_. Following this, Daniels [35] demonstrated that because of the Weibull distribution of the yarn strength, the strength of a parallel yarn bundle made of such yarns deviates from that of its constituent yarns, and acquires the value of

4.14 $$\bar{\sigma_{b}}={\left(l_{y}\alpha \beta \right)}^{-1/\beta }\exp\left(1-\frac{1}{\beta }\right).$$

It is easy to demonstrate that at given parameters *α*,*β* and sample length *l*_y_, there is always $\bar{\sigma_{y}}\geq \bar{\sigma_{b}}$, i.e. the mean yarn bundle strength cannot exceed that of its constituent yarns, as discussed in detail in [3,35].

Then, another parallel yarn bundle is introduced in the perpendicular direction and interlaced together to form a piece of fabric. If we ignore the interactions resulted, the fabric strength would be the same as the bundle strength

4.15 $$\bar{\sigma_{F}}=\bar{\sigma_{b}}={\left(l_{y}\alpha \beta \right)}^{-1/\beta }\exp\left(1-\frac{1}{\beta }\right)$$

which clearly violates the experimental data that $\bar{\sigma_{F}}\geq \bar{\sigma_{b}}$. In other words, to account for the enhanced fabric strength, one has to consider the interactions at the interlacing points when two perpendicular yarn sheets are woven together.

Here, is the key clue. It was reported that during the fracture process of both composites [24] and yarns [36] that with increasing strain on the structures, the constituent fibres break repeatedly, termed as the process before overall system failure. This phenomenon indicates that a broken fibre can again build up tension, carry load, break into even shorter segments and successively contribute towards the overall system strength. On the other hand, because of the length–strength dependency, the strengths of these fibre segments will become higher with decreasing length, thus explaining the higher systems strengths resulting.

Shahpurwala & Schwartz [3] therefore concluded that a more realistic prediction is obtained if the fabric is considered as a bundle of yarn segment whose length, termed as effective or critical length, is much shorter than the original yarn length. By back calculation using the weakest-link scaling based on the known fabric and yarn strength distributions, they determined the critical lengths, much shorter than the original yarn length and depending on the type of fabrics. Their study thus confirmed that the mechanical behaviour of a yarn in a fabric differs considerably from its *ex situ* performance tested in isolation from the fabric. However, their approach here is a more or less empirical one, thus failing to establish a theoretical relationship between the critical sub-bundle yarn length and the interactions between yarns in a tensioned fabric.

In summary, the interactions at the interlacing points have reinforced the system so much that the ultimate fabric strength is elevated from the much lower level of yarn bundles into a value sometimes even higher than that of its original constituent yarns.

Based on the model proposed by Shahpurwala & Schwartz [3] that a fabric be treated as a system of chains formed by yarn sub-bundles whose length is equal to the so-called critical length *l*_c_<*l*_y_, Pan [4] proposed that the actual mean fabric strength be calculated as

4.16 $$\bar{\sigma_{F}}={\left(l_{c}\alpha \beta \right)}^{-1/\beta }\exp\left(1-\frac{1}{\beta }\right).$$

The key now is the determination of the effective critical breaking length *l*_c_.

According to Wimolkiatisak & Bell [37], any yarn fragment with length longer than *l*_c_ is still able to break somewhere along its length as its stress exceeds its *instantaneous* *in situ* strength. So, the next length of the fragments actually varies in the range of [0.5*l*_c_–*l*_c_], with the mean length being 3/4*l*_c_. Therefore, the mean length before yarns break into *l*_c_ will be 4/3*l*_c_. Based on all of this, Pan [4] derived the theoretical expression for *l*_c_ to be used in equation (4.16) to determine $\bar{\sigma_{F}}$:

4.17 $$l_{c}={\left[\frac{1}{n_{y}\tau_{y}}{\left(\frac{4}{3}\alpha \right)}^{-1/\beta }\left(1+\frac{1}{\beta }\right)\right]}^{\beta /\beta +1},$$

where *n*_y_ is the fabric count (number of yarns per fabric length). Bringing the unit constraint *τ*_y_ from equation (4.9) into equation (4.17) one can evaluate the critical yarn length which is a function, among others, of extensions *σ*_L_, *σ*_T_, fabric weave structure *ϕ*, *t*_y_ and *n*_y_, yarn–yarn friction *μ* and yarn strength parameters *α* and *β*.

In connection to the yarn pullout analysis, the *in situ* yarn strength, i.e. the fabric strength per yarn, $\bar{\sigma_{F}}$ can be related to the yarn pulling out force in equation (4.12) as

4.18 $$\bar{\sigma_{F}}=\tau_{y}\text{Round}[n_{y}l_{c}]=P_{yu}.$$

Note that when using the unit of N yarn^−1^, the fabric tensile breaking load and fabric strength converge to the same meaning; therefore, no distinction is made between the two in our analysis.

## 5. Experimental results

To validate the theoretical predictions above and provide parameters needed for the modelling work, we also conducted the following experimental work.

### 5.1 Yarn pullout data

For the three fabrics detailed in table 1, yarn pullout tests were done only on the warp yarns. The sample preparation and pulling out tests are detailed in [29,30], and table 8 holds the test data. It is seen from the data that first when the embedded yarn length is below the critical length, the yarn will be pulled out rather than broken. In the case of the glass fabric, the yarn is so strong that even if we extended the embedded yarn length to the maximum level allowable by the machine of 220 mm, it is still below its critical length, so all yarns were pulled out. For the other two fabric types, when the embedded yarn length was increased respectively to 50 (wool) and 100 (polyester/cotton), the yarns were indeed fractured inside.

**Table 8.** The warp yarn pullout test data. Numbers in parentheses are the mean values.

fabric type	embedded length (mm)	max load (N)	*τ*_y_ (10^−1^ N per contact point)		status
polyester/cotton	50	1.45	0.13		pulled out
	70	2.16	0.14	(0.13)	pulled out
	100	2.82	0.13		breakage
wool	30	2.65	0.29		pulled out
	50	3.18	0.21	(0.23)	breakage
	100	2.59	0.18		breakage
glass	70	1.35	0.08		pulled out
	100	1.68	0.07	(0.067)	pulled out
	220	2.64	0.05		pulled out

In addition, when we introduced the yarn unit point resistance *τ*_y_, we made no differentiation yet as to whether it is for the case of yarn pulled out or broken. By looking at the data in table 8, we found that, regardless of yarn breakage or not, the *τ*_y_ values acquired appear in the same order of magnitude. Further analysis and discussion on the *τ*_y_ values are provided below.

### 5.2 Tensile strengths of yarn, yarn bundle and fabric

Next, we conducted tensile strength tests, all at gauge length of *L*=100 mm, of individual yarns, yarn bundles (to supplement the work by Shahpurwala & Schwartz [3]), and fabrics, and the results are provided in tables 9–12. For comparability, we unravelled the weft yarns from a fabric sample to test the warp yarns only as the yarn bundles, and then tested the warp yarns individually as the single yarn samples. We then compared the related strength data summarized in table 12 of all three types of samples, and confirmed from the actual data that:

(a) of the three strength values, the mean strength of yarn bundles is indeed the lowest of the three, $\bar{\sigma_{F}}\geq \bar{\sigma_{b}},\bar{\sigma_{y}}\geq \bar{\sigma_{b}},$ for all three fabrics;
(b) the mean fabric strength can be smaller than (for wool and polyester/cotton fabrics), equal to or greater (for glass fabric) than those of its constituent yarns; and
(c) if comparing the tensile strength in table 12 with the pullout data in table 8, ignoring the unbroken glass fabric, the pullout breaking force *P*_*l*m_ can be greater (wool) or smaller (polyester/cotton) than any of the corresponding yarn, bundle and fabric strengths. Besides experimental errors, which were carefully minimized during our tests, this may be an indication that *some differences in failure type may exist between the pullout test and the normal tensile test.*

**Table 9.** The tensile strength of single yarns in fabric (*L*=100 mm).

	N per yarn	
fabric type	warp	weft
polyester/cotton	3.87	2.55
wool	2.46	2.12
glass	3.98	3.02

**Table 10.** The tensile strength of warp yarn bundles (*w*=30 mm, *L*=100 mm).

fabric type	N per bundle	N per yarn
polyester/cotton	225.88	3.42
wool	204.28	2.12
glass	189.95	2.64

**Table 11.** The tensile strength of fabrics.

		N per sample		N per yarn	
fabric type	length (mm) × width (mm)	warp	weft	warp	weft
polyester/cotton	100×30	240.50	184.42	3.64	2.79
wool	100×30	222.03	213.00	2.31	2.37
glass	100×20	227.95	177.98	4.79	3.70

**Table 12.** Experimental strengths (warp only, *L*=100 mm, N per yarn).

case	single	bundle	fabric
polyester/cotton	3.87	3.42	3.64
wool	2.46	2.12	2.31
glass	3.98	2.64	4.79

## 6. Determination of the parameters

There are several parameters required in modelling that have to be determined from the experimental data.

### 6.1 The Weibull parameters *α* and *β*

We statistically estimated the values for the yarn shape and scale parameters *α* and *β* listed in table 13 for the three different fabrics based on their yarn strengths, along with the fibre density values. Then, in table 14, we provide some assumed structural values shared by all three different fabric samples, for modelling purposes.

**Table 13.** Derived parameters for calculation (warp only, *L*=100 mm, N per yarn).

case	yarn strength (N per yarn)	shape parameter *β*	scale parameter *α* (N^−*β*^mm^−1^)	fibre density (g cm^−3^)
polyester/cotton	3.87	12.71	5.80 ×10^−10^	1.43
wool	2.46	10.56	8.48 ×10^−8^	1.32
glass	3.98	7.57	1.01 ×10^−7^	2.54

**Table 14.** Assumed parameter values in calculation.

property	typical value	unit	note
fabric fibre volume fraction *v*_fy_	0.5		
yarn cross-section ellipticity *e*_y_	1.4	*w*_y_/*t*_y_	
length of yarn contact area *c*_y_	*l*_y_/2	mm	plain weave

### 6.2 Other geometrical parameters

Yarn thickness *t*_y_ can be calculated from the given yarn count

6.1 $$t_{y}(\mathrm{cm})=\frac{2}{1+e_{y}}\sqrt{\frac{\text{tex}}{\pi \rho_{f}V_{\mathrm{fy}}\times 10^{5}}},$$

where tex is the yarn count number (g km^−1^); *e*_y_=*w*_y_/*t*_y_ the yarn cross-section ellipticity; *V*_fy_ the fibre volume fraction in the yarn; and *ρ*_f_ the fibre specific density (g cm^−3^). Yarn width *w*_y_ is

6.2 $$w_{y}=e_{y}t_{y}.$$

The circumference of the yarn is

6.3 $$L_{y}=1.5\pi (t_{y}+w_{y})-\sqrt{t_{y}w_{y}}.$$

So that the length of the yarn–yarn contact area *C*_y_=*L*_y_/2 in equation (4.9).

### 6.3 The contact resistance *τ*_y_ and the critical yarn length *l*_c_

To explain the reinforcing effect of the yarn interlacing in a fabric, and to calculate the critical length *l*_c_, the contact point resistance *τ*_y_ is clearly the key parameter to be determined first. There appear multiple ways, termed below as methods 1–4, of deriving *τ*_y_ value:

(1) from equation (4.9), directly calculate *τ*_y_;
(2) from the pullout data and equations (4.12) or (4.18), for both *l*_c_ and *τ*_y_;
(3) from the bias tensile data and equation (4.18), for both *l*_c_ and *τ*_y_; and
(4) from the normal uniaxial tensile test data and equations (4.16) and (4.17), for both *l*_c_ and *τ*_y_.

It has been demonstrated in [4] how to obtain *τ*_y_ value using method 1, involving many parameters including both stresses *σ*_*L*_ and *σ*_*T*_. A thorough analysis on it is available there. We will therefore not pursue it further. If we look at the three types of experiment, i.e. the normal tensile test, the bias tensile test and the yarn pullout, there are really two distinctive events individually or jointly leading to the failure of a fabric sample, namely the yarn pullout and yarn breakage. These two events thus represent two different fabric failure mechanisms or types. We term them *type I* in yarn pullout, and *type II* in yarn breakage. As a result, we can classify the test methods in terms of the failure types involved, such as the yarn pullout test (type I dominant, corresponding to method 2 in the above list), normal tensile test (type II dominant, method 4) and the bias tensile test (type I + type II, method 3). *If we want to reflect the distinctive impact on fabric structure of the two failure types, it is only reasonable that we use different τ*_y_ (*thus l*_c_) *values in predicting the corresponding test results.*

The actual calculation is for the polyester/cotton fabric only. For method 2 of yarn pullout test, we can get the data from table 15 based on equation (4.18), *τ*_y_=0.0132 N per point and *l*_c_=6.0 cm, averaged over the pullout results. For method 4 of normal tensile test, it is shown in table 12 that $\bar{\sigma_{F}}=3.64\;\text{N per yarn}.$ Plugging it first into equation (4.16) gives *l*_c_=3.83 cm, and then *l*_c_ into equation (4.17) to find *τ*_y_=0.063 N per point.

**Table 15.** *τ*_y_ and *I*_c_ values determined via different methods (polyester/cotton, warp only).

case	method 2 pullout	method 3 bias	method 4 normal
*τ*_y_ (N per point)	0.0132	0.091	0.063
*I*_c_ (cm)	6.00	1.19	3.83

**Figure 5.**
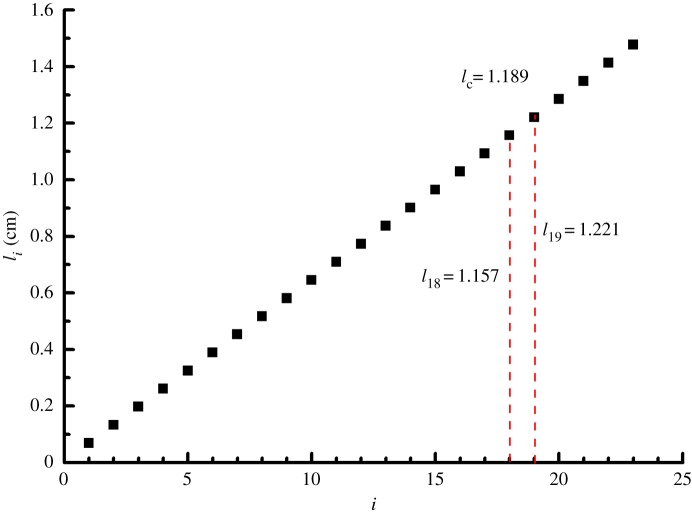
Distribution of embedded yarn lengths and the critical value *l*_c_.

As for method 3, however, some additional steps are involved to derive the critical yarn length *l*_c_. Take the bias tensile test results in table 5 for polyester/cotton sample. During the test, all yarns were pulled out when sample width is low. Yarn breakage occurred only when sample width *W*=15 mm with the total yarn number *P*=23, there are *M*=18 yarns pulled out, named yarns 1,2,…,18, and *N*=5 broken, named 19,20,…,23. Using equation (4.1) for yarns *i*=1–23, we can calculate the embedded yarn length distribution in figure 5. As the longest 5 will be broken, then the critical yarn length should be between the lengths for yarns 18 and 19, and a simple average gives *l*_c_=1.189=(1.157+1.221)/2. Then, from the bias test results in table 2, the sample breaking load is 43.96 N when *W*=15 mm. There are 5 yarns broken, carrying a total of 5×3.64=18.2 N, where the *in situ* yarn strength, i.e. the fabric strength per yarn, $\bar{\sigma_{F}}=3.64$ is from table 12. So, the total 18 slipping yarns contributed 43.96−18.2=17.47 N force, which translates via equation (4.18) into *τ*_y_=0.091 N per point.

In the end, all *τ*_y_ values are listed in table 15 from methods 2–4 for polyester/cotton sample, confirming that the values derived using different methods indeed differ quite significantly, and the ranking of the *τ*_y_ values is method 3 (bias)> method 4 (normal)> method 2 (pullout). That is, in a bias sample, the resistance at interlacing point is intensified because of the obliquity of the yarns.

## 7. Predictions and comparison with the experimental data

### 7.1 Single yarn, yarn bundle and fabric strength calculation and comparison

For verification of the results obtained so far, we used *α* and *β* values for all three fabrics to calculate the individual yarn strength using equation (4.13), and yarn bundle strength using equation (4.14). All the results in comparison with the corresponding experimental data are provided in table 16.

**Table 16.** Strengths for different cases (warp only, N per yarn). e, experiment; p, prediction. Data used: method 4 for *τ*_y_ and *I*_c_.

case	single $\bar{\sigma_{y}}$	bundle $\bar{\sigma_{b}}$	fabric $\bar{\sigma_{F}}$	contact resistance (N per point) *τ*_y_	critical length *l*_c_ (cm)
polyester/cotton^e^	3.87	3.42	3.64		
polyester/cotton^p^	3.56	3.36	3.64	0.063	3.83
wool^e^	2.46	2.12	2.31		
wool^p^	2.88	2.73	2.31	0.0018	58.77
glass^e^	3.98	2.64	4.79		
glass^p^	4.29	4.15	4.79	0.095	3.41

It is seen from table 16 that although variations do exist, we nonetheless confirmed that the mean strength of yarn bundles, both experimental and predicted values, is the lowest of the three, $\bar{\sigma_{F}}\geq \bar{\sigma_{b}},\bar{\sigma_{y}}\geq \bar{\sigma_{b}},$ for all fabrics, except fabric 2 (wool) whose predicted bundle strength is greater than its fabric strength.

For the normal fabric strength prediction from equation (4.16), we should use the corresponding value of critical length *l*_c_ from method 4. The *l*_c_ and *τ*_y_ values for the other two, wool and glass, fabrics are also calculated using the same method 4, also shown in table 16. It is interesting to see that both *l*_c_ values for fabric 1 (polyester/cotton, *l*_c_=3.83 cm) and fabric 3 (glass, *l*_c_=3.41 cm) are smaller than the original length *L*=10.0 cm. But, the value for fabric 2 (wool, *l*_c_=58.77 cm) is larger than 10.0 cm, indicating that the fragmentation-invoked reinforcing effect is not significant enough in fabric 2 that its fabric strength $\bar{\sigma_{F}}$ is lower than its bundle strength. Conversely, for the other two fabrics their *l*_c_ values are smaller than the original 10.0 cm, so that their fabric strengths are indeed enhanced.

### 7.2 Numbers of total, broken yarns as a function of sample width *W*

Again take the polyester/cotton fabric for example. As we are dealing with the bias tensile test involving yarn pullout, we use the *τ*_y_ and *l*_c_ values calculated via method 3 in table 16. The fabric account in the warp direction is 22 cm, so that for a given sample width *W*, the corresponding total number of yarns can be calculated from equation (4.1), as shown in table 17.

**Table 17.** Bias test results as functions of sample width *W* (polyester/cotton). e, experiment; p, prediction. Data used: method 3, *τ*_y_=0.091 (N per point), *l*_c_=1.19 (cm).

*W* (mm)	5	8	10	15	20	25
total yarns	9	13	16	23	32	40
broken	0	0	0	5	14	22
pullout	9	13	16	18	18	18
sample strength^p^ (N)	5.82	11.74	17.47	35.67	68.43	97.55
sample strength^e^ (N)	1.39	4.44	17.40	43.96	87.03	104.05

**Figure 6.**
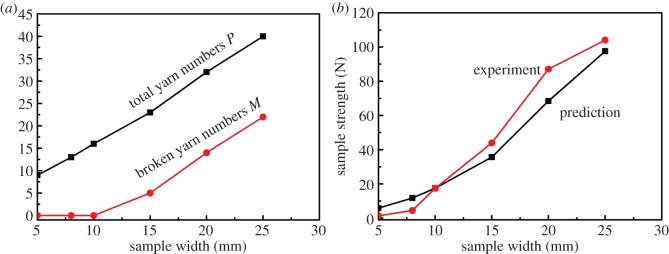
(*a*) Predicted numbers of broken and total yarns in sample widths. (*b*) Predicted and experimental sample strengths versus widths.

For a sample of given width *W* and total yarn number *P*, the embedded yarn lengths distribution (rounded to integer) can be calculated from equation (4.1), as illustrated in figure 5. Note that for a given fabric, its critical length *l*_c_ is a fixed fabric property. In the beginning when sample width *W* is so small whose longest embedded yarn length *l*_*i*_<*l*_c_, so all the yarns in the sample will slip. As the sample width *W* increases, the embedded yarn lengths grow. As soon as the sample width reaches a critical value, so that there is a yarn with embedded length *l*_*i*_≥*l*_c_, the yarn breakage takes place. Beyond this critical sample width, as the number of pullout yarns *N* remains constant, the other *P*−*N*=*M* yarns are the broken ones. So, in table 17 for the polyester/cotton fabric, *l*_c_=1.19 cm, the numbers of pullout and broken yarns are calculated corresponding to each given sample width *W*, and plotted in figure 6*a*. The parallelism between the lines of total and broken yarn numbers in figure 6 suggests that once yarn breakage starts, as both specimen width *W* and total yarn number *P* grow, the number of slipping yarns *M* stays nearly the same, whereas the broken yarn number increases. Figure 6*a* is in good agreement with the corresponding experimental results in figure 3.

### 7.3 The breaking load of bias samples as a function of sample width *W*

A fabric sample, bias at *θ* and with *M* broken and *N* slipping yarns during the test, will achieve a total breaking load *P*

7.1 $$P=P_{M}+P_{N},$$

where the contribution from all *M* broken yarns is

7.2 $$P_{M}=M\bar{\sigma_{F}}.$$

Again, the *in situ* yarn strength, i.e. the fabric strength per yarn $\bar{\sigma_{F}}=3.64\;\text{N per yarn}$ in table 16 calculated from equation (4.16), is used here. The contribution from all *N* slipping yarns is

7.3 $$P_{N}=\sum_{i=1}^{N}\text{Round}[n_{y}l_{i}]\cdot \tau_{y},$$

where the *τ*_y_=0.091 N per point from method 3 corresponding to the bias tensile test is used in calculation. The embedded yarn length *l*_*i*_ for the slipping yarns can be determined using the combination of equations (4.2) and (4.3).

As *M* and *N* are functions of fabric width *W*, the total breaking load of the fabric sample thus also becomes a function of fabric sample width. The total bias sample strength at each width is calculated also in table 17, in comparison with the corresponding experimental results. The values then are plotted in figure 6*b*, and a consistent general trend between the data of the two groups is apparent. Examining the data in table 17 in more detail, for narrow width samples where yarn pullout is dominant during a test, the predicted sample strength is greater than the experimental data. This continues until *W*=10 mm just before yarn breakage takes place, where the two results coincide. Once beyond that sample width, the predicted results are below the experimental ones.

One explanation for the overestimation in the pure yarn slippage stage is that we implicitly assumed the contact resistance *τ*_y_=0.091 N per point remains constant along an embedded yarn length *l*_*i*_, but this is unlikely the case and the resistance may be smaller at both yarn ends. Of course, the variations in fabric structure and properties, and, especially, the distortion in fabric structure during the test are all other possible contributors to the errors in predictions.

## 8. Conclusion

This is the first complete study in exploring the origin of the tensile strength of a narrow and bias woven fabric via theoretical analysis and experimental validation. Furthermore, the conclusions drawn can be helpful in understanding the failure process of fabric-reinforced composites where the larger reinforcement breaks down to small pieces, yet still carrying load and contributing collectively to the overall behaviours of the system. More importantly, this experimental and modelling work actually presents a methodology dealing with the fracture mechanics of a discrete system formed by components associated to each other only via contact friction.

More specifically, the frictional interactions between the warp and weft yarns in a fabric are essential to the fabric mechanics and such frictions provide both connectivity and mobility to the entire fabric structure. The interlacing point resistance *τ*_y_ is thus the key parameter in reflecting the intensity of such interaction.

Compared with the uniaxial normal tensile tests, bias extension tests can capture more information in understanding the complex mechanics in woven fabrics. The resultant tensile strength in a bias sample involves both yarn pullout and breakage, and the relative proportion of the two failure types depends on, as demonstrated in this study, the bias direction angle *θ*, sample width *W* and length *L*, along with other factors known to affect fabric strength tested in principal directions. A bias tensile process can hence be considered a mixed type of the yarn pullout test (type I) and yarn breakage (type II), and the *τ*_y_ resistance acquires different values for different sample failure types. Consequently, in theoretically predicting the behaviour of a bias tensile sample, the appropriate value of the point resistance *τ*_y_ has to be used in modelling each type. Furthermore, even for a single type, the *τ*_y_ value may not be a constant along the entire embedded yarn length, especially in yarn pullout test where variation in *τ*_y_ value seems to be more realistic.

In addition, the critical embedded yarn length *l*_c_ can be used as a criterion to judge the existence and degree of the fragmentation-invoked *in situ* reinforcing effect in a fabric.

## Data accessibility

Deposited in DataDryad.org (http://doi.org/10.5061/dryad.5km60), including: the original data for all three samples: (file folder) dataforallthreesamples/. Additional information for tables 3 and 4: (file) InforTables3&4.docx.
